# Democratizing Microreactor Technology for Accelerated Discoveries in Chemistry and Materials Research

**DOI:** 10.3390/mi15091064

**Published:** 2024-08-23

**Authors:** Tomomi Sato, Koji Masuda, Chikako Sano, Keiji Matsumoto, Hidetoshi Numata, Seiji Munetoh, Toshihiro Kasama, Ryo Miyake

**Affiliations:** 1 Graduate School of Engineering, The University of Tokyo, Kawasaki 212-0032, Japan; kasama@g.ecc.u-tokyo.ac.jp (T.K.); trmiyake@mail.ecc.u-tokyo.ac.jp (R.M.); 2 Department of Physics and Astronomy, University of Exeter, Exeter EX4 4QL, UK; k.masuda@exeter.ac.uk; 3 IBM Semiconductors, IBM Research–Tokyo, Kawasaki 212-0032, Japan; chikako.sano@ibm.com (C.S.); keim@jp.ibm.com (K.M.); hnumata@jp.ibm.com (H.N.); munetoh@jp.ibm.com (S.M.)

**Keywords:** microreactor, accelerated discovery, flow chemistry, advanced integrated microreactor development platform, machine learning, artificial intelligence

## Abstract

Microreactor technologies have emerged as versatile platforms with the potential to revolutionize chemistry and materials research, offering sustainable solutions to global challenges in environmental and health domains. This survey paper provides an in-depth review of recent advancements in microreactor technologies, focusing on their role in facilitating accelerated discoveries in chemistry and materials. Specifically, we examine the convergence of microfluidics with machine intelligence and automation, enabling the exploitation of the cyber-physical environment as a highly integrated experimentation platform for rapid scientific discovery and process development. We investigate the applicability and limitations of microreactor-enabled discovery accelerators in various chemistry and materials contexts. Despite their tremendous potential, the integration of machine intelligence and automation into microreactor-based experiments presents challenges in establishing fully integrated, automated, and intelligent systems. These challenges can hinder the broader adoption of microreactor technologies within the research community. To address this, we review emerging technologies that can help lower barriers and facilitate the implementation of microreactor-enabled discovery accelerators. Lastly, we provide our perspective on future research directions for democratizing microreactor technologies, with the aim of accelerating scientific discoveries and promoting widespread adoption of these transformative platforms.

## 1. Introduction

### 1.1. The Era of Accelerated Discovery

The rapid adoption of artificial intelligence (AI) technologies, notably large language models, has ushered in an era of accelerated discovery (AD) in scientific research and technology development [1]. This trend holds significant potential for transforming the fields of chemistry and materials research [2,3,4]. With an abundance of available data, including scientific literature, patents, databases, and simulation models, these disciplines are particularly amenable to leveraging AI capabilities. Using a powerful computational pipeline, a vast amount of data can be analyzed to extract patterns in the data, construct inference models, and generate hypotheses, leading to the invention of novel molecules, materials, or processes. Further incorporating laboratory automation technologies, this combined approach can significantly reduce the time and cost spent from the conception of an idea to the delivery of an outcome. Its economic impact should be tremendous, as the material and chemical industry benefits directly from accelerating cycles of innovation [5]. Furthermore, in the face of a global health and environmental crisis, scientists and technologists must address the urgent needs for effective medical treatment and sustainable development by enabling rapid and cost-effective translation of ideas into tangible solutions through the use of AI technologies [6].

**Figure 1:**
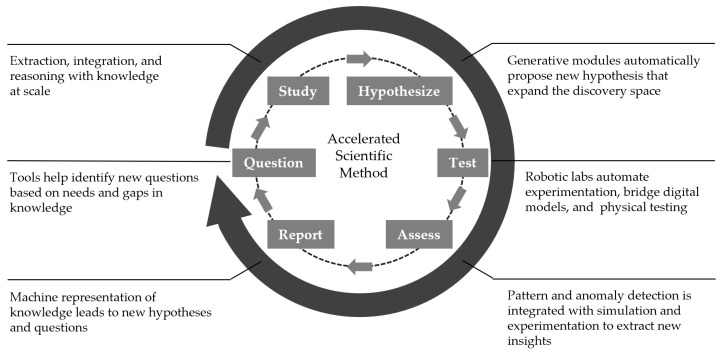
This is a general framework of accelerated discovery campaigns consisting of the creation of a knowledge base, the construction of an inference model, the generation and experimental validation of a hypothesis, and the feedback for the next search cycle.

Chemistry and materials sciences are amongst the most amenable to the data-driven discovery approach due to several factors, including the vast amount of scientific data available in various entities (e.g., scientific literature, patents, and databases) and the underlying physical rules that define molecular and material properties or determine the outcome of chemical reactions. As such, recent years have seen a surge of publications demonstrating applications of machine intelligence to accelerated discovery in chemistry and materials research and laboratory automation. Examples include optimization of small organic synthesis [7], polymer synthesis [8], nanomaterial synthesis [9], synthesis route hunting of small organic molecules [10,11], discovery of novel thin-film materials [12], and optimal formulation of 3D-printing materials [13], to name a few. These recent studies successfully demonstrated the potential of the accelerated discovery concept to cut down the idea-to-result time while also substantially reducing overall operational cost and environmental impact by minimizing material consumption in the experimental validation. With the rapid advances in machine intelligence and robotic automation, the concept of an autonomous laboratory is now demonstrated as a viable technology [14]. A general workflow of accelerated discovery consists of a knowledge base, model inference, hypothesis generation, and experimental validation, which iterates until a desired outcome is obtained (Figure 1) [15].

Despite such remarkable progress in the field, several practical challenges remain to be addressed. While there is an abundance of chemistry and materials data to be utilized, which is one of the motivations of the field and the driving force of some of the initial studies, data required to train a machine learning (ML) model for a specific task is often limited. For example, to make a prediction model for a certain physical property of a new material (e.g., a thermal property of a hypothetical polymer), similar property data for similar materials are needed. However, most of the available data come in relatively small sizes and various formats, highly depending on the material, experimental/simulation methods, lab where the data are collected, and consensus of the community of practitioners. The variability of data format and the lack of standardized data management can hamper the widespread application of AI in chemistry and materials science despite its potential. The concept of the Foundation Model, first demonstrated in the field of natural language processing [16], is to overcome the scarcity of chemical/material data for training a large and robust machine learning model. By “stitching” ML models trained for particular applications with different modalities, such as texts, X-ray images, SMILES, etc., one can create a larger model that contains abstract knowledge of a subject, such as chemistry or materials. A model for a specific task can be obtained by fine-tuning the model parameters by training with small datasets. It can be a powerful technology that can extend the capability of AI models in chemistry and materials with existing data. In addition, the experimental validation of a hypothesis generated by AI can be a laborious and time-consuming process, as it often requires the optimization, if not the whole development, of a reaction process to produce the molecule or material to be tested. Therefore, it is equally important to invest in hardware instrumentation to increase the throughput of the experimental validation. Improvement in the experimental instruments can also address the scarcity of domain-specific data for model training by increasing the rate of data generation. Robotic automation has been successful in speeding up experimental workflows and reducing human-related errors. For many chemical reactions, improvements in the liquid handling system will allow rapid and precise control of reaction conditions, such as concentration, temperature, and pressure, leading to high-throughput generation of consistent and reliable experimental data.

### 1.2. Flow Chemistry Using a Microreactor as a Platform for AD

In terms of accelerating experimental validation by improving instrumentation, flow chemistry stands out as a promising approach that has recently been developed to address the drawbacks of traditional combinatorial chemistry. Combinatorial chemistry is a technique to synthesize a wide variety of compounds following the same experimental procedures except using different combinations of materials and conditions to search for compounds with a target function and activity. It has been used extensively in the fields of organic synthesis and pharmaceutical product development [17,18], enabling the synthesis and evaluation of several hundred thousand reactions in a single experimental run using a specialized platform. However, it has potential limitations. First, due to the fixed experimental procedure that is static throughout a run, it tends to produce a large number of structurally similar compounds, which results in inefficient lead search or a low occurrence rate of hit compounds accompanied by a large amount of waste and high operational costs. Second, since, in general, chemical synthesis is a multistep and exothermic process and the decomposition of reagents affects subsequent reactions, it is desirable to extract the products from the reaction system quickly. Instruments designed for combinatorial chemistry are generally not ideal to do so.

Flow chemistry using a microreactor has recently attracted attention as a method to overcome the above-mentioned limitations of combinatorial chemistry. In particular, by using a microreactor, flow chemistry has the following advantages in contrast to batch processing:

- Rapid mixing: Rapid and uniform mixing of reactants due to shortened diffusion distance
- Precise temperature control: Efficient heat transfer due to large specific surface area
- Precise residence time (reaction time) control: Controllable residence time by reactor volume and solution flow rate

With these remarkable features, microreactors can achieve highly precise and controlled reaction conditions intractable by bulk systems. For example, even if the reaction intermediates are unstable and available only for a very short time, fast mixing in a microreactor allows the intermediates to contribute to the reaction of the target compounds in high yields while suppressing by-products. On the other hand, Microreactors, due to their use of narrow flow channels, have difficulty handling solid materials. When transporting slurry-like substances, there is a risk of solids clogging the channels. Additionally, with high-viscous fluids, flow within the channels becomes challenging, making efficient reactions difficult and increasing pressure loss. This necessitates various structures of microreactors and rapid manufacturing tools suitable for target chemical reactions. Several microreactor systems have already been commercialized and are available for some specific pharmaceutical and fine chemical productions [19,20,21]. However, the types of microreactors provided are limited, and they do not have enough versatility as a platform for AD.

**Figure 2:**
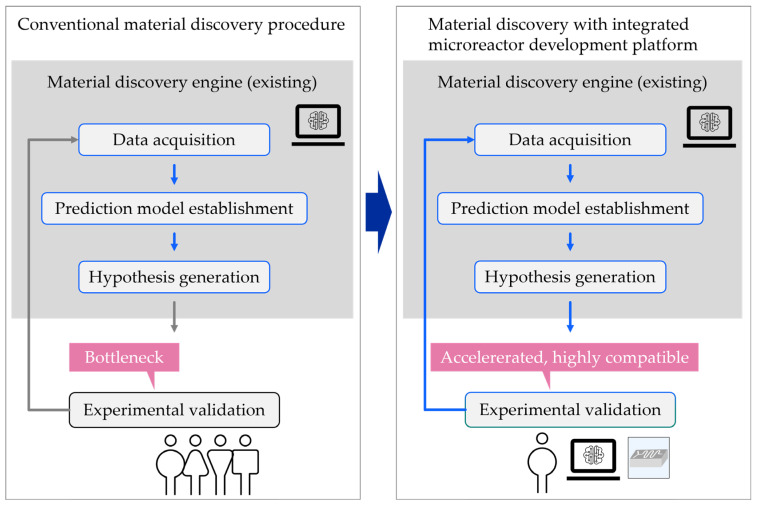
Material discovery with integrated microreactor development platform.

Building a robust and effective machine learning model for an accelerated discovery campaign system generally requires a large amount of experimental data over a variety of parameters, such as reaction conditions, including temperature, concentration, solvent types, catalyst species, and residence time. Flow chemistry using a microreactor can produce a large volume of high-quality experimental data, including data that can only be attainable using a microreactor system. A machine intelligence system trained with such data will have extended capability beyond the system trained by currently available data. We envision that a discovery accelerator system equipped with a microreactor-enabled experimental platform will further drive the advancement of chemistry and materials research ever faster and more productively. Therefore, it is worthwhile to overview the current state of the art of the microreactor technologies, especially in the context of accelerated discovery, and investigate their applicability (Figure 2).

### 1.3. Review

There are a number of excellent reviews and tutorials on various aspects of microreactor technologies, such as physical and chemical properties, applications for chemical sensing and chemical synthesis, and implementation of high-throughput experiments. While those topics are briefly reviewed and discussed, the main goal of this review is to provide the readers with an updated overview of microreactor technologies as a discovery accelerator in chemistry and materials research. We will highlight selected bodies of results demonstrating the advantages, as well as limitations, of a microreactor-enabled discovery accelerator for increasing the accuracy of measuring and controlling experimental parameters, increasing experimental throughput, and thereby improving the productivity of experiments. This review is by no means intended to be comprehensive nor to represent the consensus of microreactor communities. Its intention is rather to present the authors’ personal view of the potential and opportunities surrounding microreactor technologies and to suggest future research directions to disseminate the use of microreactors as a discovery accelerator in chemistry and materials research.

To this end, this review paper is structured as follows. In the next section, we will highlight important physical and chemical characteristics of microreactors, typical fabrication methods, and basic instrumentation techniques of a microreactor system, as well as the integration of AI and automation technologies to build AI-enabled discovery accelerator systems. In Section 3, we will highlight selected bodies of previous research work on microreactor-enabled accelerated discovery systems applied to chemistry and materials. In Section 4, we will pick out some emerging technologies that may help ease the implementation of a microreactor-enabled discovery accelerator and further extend capability. In Section 5, we will discuss future opportunities for democratizing microreactor technologies for accelerated discovery.

## 2. Elements of Microreactor-Enabled Discovery Accelerator

### 2.1. Characteristics of a Microreactor

Scaling down the physical dimensions of a reactor space can have significant effects on the fluid dynamics of flow and, thus, on the kinetics of chemical reactions. In a reaction field at a microscopic scale, a homogeneous mixing state can be quickly created by molecular diffusion. Therefore, a continuous flow process in which two fluids are mixed and reacted while continuously flowing can be used. For example, in the case of ethanol, when the solvent is water, the diffusion coefficient is 0.84 × 10^−3^ mm^2^/s (at 25 °C), so it can diffuse in as little as 3 s at 50 µm and 0.1 s at 10 µm [22,23]. The width of the channel should be less than 100 µm, which is the general dimension to bring out the effect of molecular diffusion effectively. Although there is no strict definition of a microreactor, it can be considered as a continuous flow reactor with a mixing zone of 100 µm or less in width. Even if the flow channel width is more than 100 µm, the effect of molecular diffusion can be expected if the dimensions of the internal mixing zone are all less than 100 µm [24,25,26,27]. On the other hand, as the reaction field becomes smaller, the effect of physical quantities that depend on surface area becomes relatively stronger than those that depend on volume. The effects of mass, inertia force, etc., as parameters related to volume become gentle, and the effects of viscous force, interfacial tension, heat transfer, mass transport, etc., as parameters related to surfaces and interfaces become significant. In a broad sense, reactors that make good use of these dimensional effects can also be considered a type of microreactor [28,29,30].

### 2.2. Fabrication Technologies for Microreactor

Microreactors are fabricated using well-established semiconductor technologies. Therefore, extremely accurate construction with sub-micron resolution enables highly precise control of the characteristics mentioned above [31].

In particular, chemical reactions are often performed by connecting corrosive materials under high-temperature and high-pressure environments. Therefore, resins and silicone elastomers often used in ordinary analytical devices may not be durable enough to handle such reactions. Then, durable materials such as glass or metal are often selected from the viewpoints of chemical resistance, heat resistance, and robustness. In addition to general lithography and etching techniques, precision machining techniques such as wire electrical discharge machining and high-precision cutting are often used to form structures such as fine flow channels. As a recent example, a discussion on the deployability of glass is given in the case of a technique in which a metallic mold (tungsten carbide-cobalt) with fine convexity machined by wire EDM is pressed against a glass plate and imprinted to form glass channels under high temperatures [32]. The glass is also highly resistant to corrosion and effective as a catalyst. In another example, a stainless steel microreactor was formed by precision machining using Hastelloy, which is highly resistant to corrosion and is expected to be effective as a catalyst as an inner wall material. Moreover, a reactor was formed by sintering SiC with excellent thermal conductivity onto a Si structure created by microfabrication [33]. Each fabrication technique requires suitable materials and organized processing methods that are appropriate for each chemical reaction type, taking temperature, corrosion, and chemical resistance into consideration.

**Figure 3:**
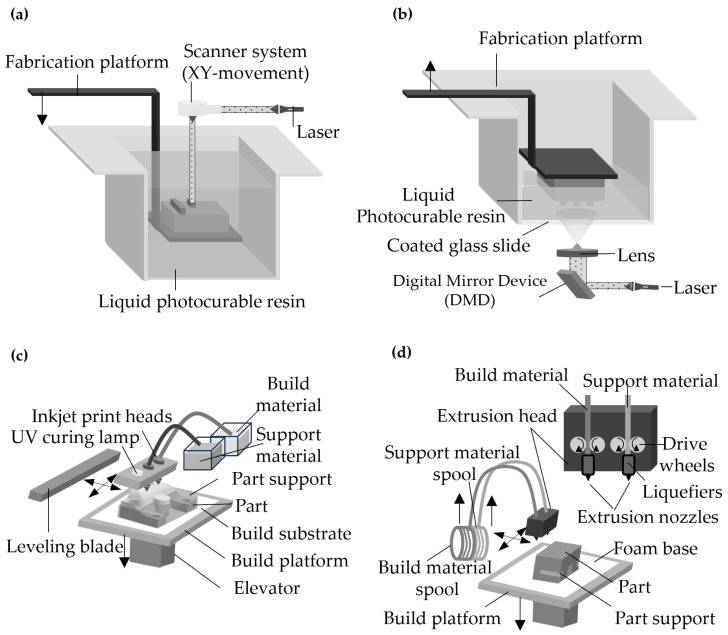
Four 3D printing configurations. (**a**,**b**) stereo-lithography apparatus type 3D printer and digital light projection type 3D printer. (**c**,**d**) multiple-jet modeling type 3D printer and fused deposition modeling type 3D printer.

To make various structures of microreactors rapidly, in recent years, there have been many attempts to apply 3D printing technologies to produce microreactors (Figure 3).

Ko et al. summarized recent achievements in micro-reaction modules and micro-separation units [34]. They looked into recent developments of micro-reaction systems fabricated by various 3D printing techniques for chemical synthetic applications.

Sercer et al. investigated the methodology of producing a 3D-printed microreactor from the acrylic resin by the PolyJet Matrix process [35].

The production of microreactors by the PolyJet Matrix technology comes with some benefits compared to traditional machining. A microreactor can be easily constructed by computer-aided design and transferred to the 3D printer, which reduces the development time of a microreactor prototype. Also, a low cost of construction materials is important when considering multiple design variations for different prototypes.

Scotti et al. investigated a simple flow chemistry microreactor with an electrospray ionization tip for real-time mass spectrometric reaction monitoring [36]. The microreactor was fabricated using a laser-based additive manufacturing technique from acid-resistant stainless steel 316 L. The functionality of the microreactor was investigated by using an inverse electron demand Diels-Alder and subsequent retro Diels-Alder reaction for testing. The microreactor is potentially useful as a disposable device.

Emerging technologies of 3D printing have the potential to realize vertically stacked microchannels and miniaturization of bulky micro-reaction accessories. The microreactor manufacturing technologies are summarized in Table 1, along with their applications, characteristics, and corresponding reference numbers.

### 2.3. Online Reaction Monitoring

For efficient reaction parameter survey and pathway exploration using microreactors, it is important to rapidly measure the environmental conditions and reaction generation conditions at the reaction site. Generally, a method to measure the concentration of reaction products is to collect a sample containing reaction products downstream of the reactor and introduce it into an analyzer for quantitative analysis or to install a sensor in the downstream flow channel to detect the product concentration in order to approach continuous measurement. However, it is necessary to consider that the concentration and temperature environment may change under flow conditions from the reactor to the place of detection and that the product composition may change accordingly. Therefore, many methods have been proposed to directly and continuously observe chemical reactions and product formation in the reaction site field inside the reactor to more accurately understand the characteristics of microreactors, the high responsiveness and homogeneity of reaction conditions, and the effect of the high controllability of reactions resulting from these conditions. One method is to mount a sensor inside the reactor for direct observation, and the other is to access the reaction event field from outside the reactor in a non-contact manner. The former includes an example of a thin-film electrode that serves as a thermocouple on the inside wall of the reactor [37], an example of a flow velocity sensor that can be operated even when installed in a highly corrosive liquid [38], and a unique example of a resonant cantilever installed inside the reactor to observe thermal weight changes from the outside using TEM [39]. In any case, however, there are concerns about the durability of the sensor installed inside the reactor and its own influence on the reaction in a reaction field environment. In contrast, there are many reports on the latter method of non-contact observation of reaction conditions from outside the reactor. However, in all examples of this method, it is necessary to form a light-transmitting window in the reaction field, which limits the shape and materials of the reactor, and the target reactions are restricted by the characteristics of the materials. Given its applicability to a wide variety of chemical reactions, many examples of X-ray absorption and nuclear magnetic resonance have been reported since 2000 to observe reaction processes in microreactors composed of inorganic and metal materials. X-ray absorption is often used to target metals as products, for example, to observe the growth process of metallic nanoparticles [40]. However, to increase the contrast, some efforts have been made to make the reactor wall of the X-ray transmitting part thinner and to lengthen the exposure time. In the case of nuclear magnetic resonance, which is advantageous for the synthesis of organic materials, the flow channel shape of the microreactor is made thinner, and a planar coil is integrated directly above the flow channel. As a result, the permeability is improved, the signal intensity increases, and the sensitivity is enhanced [41,42,43]. There are optical methods of product detection, such as infrared and Raman spectroscopy [44]. Table 2 summarizes the reaction monitoring introduced. However, at present, a sensitivity that exceeds that of off-line devices has not been achieved. Although various methods have been proposed to directly observe reaction processes in microreactors, it is necessary to select an observation method that takes into account the material properties of the target reaction and the state of the products and to prepare the reactor shape, dimensions, and materials individually accordingly.

### 2.4. Device Design Tools

The prosperity of the semiconductor industry over the past decades would not have been possible without electronic design automation (EDA) tools. EDA tools have enabled efficient and reliable circuit design to verify this scale, instead of fully manual circuit design done by humans. As an input for EDA tools, a hardware description language (HDL) represented by Verilog HDL or VHDL can be used to express the circuit’s structure and behavior.

A similar concept exists in microfluidic chip design; computer-aided design tools are being developed for microfluidic large-scale integration, or mLSI for short. Conventionally, microfluidic device design was done manually using software such as SolidWorks or AutoCAD, based on simulation and verification with software such as MATLAB or COMSOL. Research has been done to replace manual design with semi- or fully automated physical design iteration Mausing MDA tools, along with the development of a microfluidic hardware description language (MHDL) that specifies architectures of the chips as an HDL does for semiconductor circuits.

In 2013, McDaniel et al. [45] introduced an MHDL to define the structural specifications of devices with a syntax based on VHDL. This MHDL handled the description of the predefined components and the interconnection of the components, along with the entities. Their MHDL compiler converted the MHDL device specifications into a netlist, which is a graph-based data structure that describes the connection of the components.

In 2019, Sanka et al. [46] introduced an MHDL named MINT, and an MDA interchange format named ParchMint. MINT separated structural and behavioral aspects of the design, which enabled generic designs that were not tied to a specific assay or netlist. While component representations in previous research had fixed specifications, MINT allowed adjustable geometric parameters that defined its size, performance, and functional characteristics.

**Figure 4:**
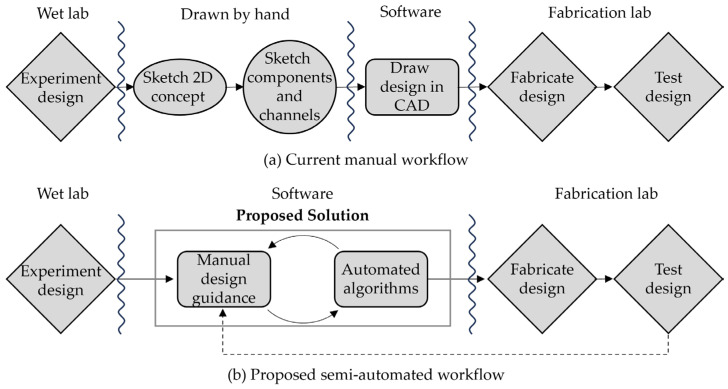
Current manual (**a**) and proposed semi-automated (**b**) workflows for mVLSI LoC design. CAD algorithms can augment the semi-automated workflow, accelerating the design process without replacing it entirely.

Based on interviews with ~100 LoC designers, McDaniel et al. [47] developed a semi-automated mVLSI drawing tool (Figure 4). They mentioned that the designers’ primary concern was the rapid acquisition of a working device with a minimum amount of prototyping, with no trust and no interest in fully automated “push-button” end-to-end solutions. Therefore, they declared that there was a need for a semi-automated design method with (1) faster algorithms capable of performing smaller discrete steps in seconds or minutes, (2) algorithms that generate partial solutions that do not alter user-specified design constraints, and (3) algorithms based on the inputs provided by an expert designer, instead of an ideal set of inputs obtained in a fully automated design flow. Their suggestion was that the objective should be to provide guidance to the designer rather than replacing the existing design process. Their semi-automated drawing tool was based on a collection of mVLSI components stored in a library of entity files described with a structural netlist specification language such as an MHDL or MINT. The placement was performed manually by default, although full or semi-automated routing solutions were available.

Sanka et al. introduced 3DµF, a web-based interactive design tool that integrated a wide assortment of engineering techniques used in the design of microfluidic devices [48]. It incorporated features in design techniques such as identification of functional units and procedural component generation, along with design automation such as automatic transformation of different layers, manufacturing the optimized effort, and realization of a standard microfluidic component library.

Recently, Lashkaripour et al. introduced DAFD, a web-based microfluidic design automation software with machine learning [49]. Focusing on droplet generators, their machine learning algorithm converted the user-specified performance, droplet diameter, and generation rate into the required geometry and flow rates. They mentioned that by combining DAFD’s parameter exploration with 3DµF’s design abilities, a fully realized design can be created rapidly without going through monolithic designs where both the structure and the function are encoded in a non-modifiable manner.

### 2.5. AI and Robotics

ML techniques have been actively applied in the field of microreactors to improve the productivity of experiments, such as in throughput, reproducibility, and product yield, making ML quickly an important element of a microreactor system.

ML techniques are used to predict experimental outcomes for given reaction conditions or to optimize experimental parameters to reach a desired output. Predictions of outcomes from microreactor experiments are based on a prediction model built on data from previous experiments or simulations. Optimization refers to the overall, often autonomous, optimization of a closed-looped experiment planning. Compared to prediction and optimization, generalization refers to experiments where effective parameters are rather unknown and thus need to be specified.

For predictive tasks, commonly used ML algorithms are families of artificial neural networks (ANNs), such as convolutional neural networks (CNN) and recurrent neural networks (RNN). Lack of training data obtained from physical or numerical experiments often limits the applicability of ANNs for prediction tasks in many flow chemistry studies.

Coley et al. demonstrated a flow synthesis robotic platform with NN models integrated within the modular components [11]. For example, in the synthesis-planning module, a feedforward NN model was trained to predict which of the transform rules were most applicable to a target molecule based on its molecule structure. Rizkin et al. used NNs to predict the molecular weight, polydispersity, and reactor temperature from the reaction space of a polymerization reaction [50]. In their subsequent research, they demonstrated that their machine learning approach leads to efficient polymerization design under automated microfluidic experimentation.

Optimization methods can be classified into frequentist methods, represented by Reinforcement Learning (RL), and Bayesian methods, represented by Bayesian Optimization (BO). The former method approaches unknown factors decisively, while the latter approaches them stochastically.

Meanwhile, to identify the most effective methods for accelerated materials development with multiple objectives, Epps et al. have conducted simulated autonomous materials development experiments using a multistage surrogate material synthesis model integrated with AI-guided decision-making strategies within a single-period horizon reinforcement learning algorithm [51]. In particular, the ensemble neural network-based decision-making algorithm enabled more efficient material formulation optimization in a no-prior-information environment.

BO methods are derivative-free global stochastic optimization methods that are particularly well-suited for expensive-to-evaluate problems. Schweidtmann et al. used BO algorithms to identify a set of optimal conditions corresponding to the trade-off curve between environmental and economic objectives of chemical reactions to find environmentally acceptable and economic operating conditions for the flow [52].

Other research suggests combining different prediction methods for optimization. Mekki-Berrada et al. suggested that this can realize ML frameworks that can drive high-throughput experimental platforms from the very beginning of the screening process where the dataset is sparse to more resolved screening states where the dataset is larger [53]. BO can efficiently explore the parameter space and target specific material properties under a sparse dataset. However, their disadvantage lays in the fact that they do not give general insights into the reaction process and also in the fact that the performance depends on the initial choice of model hyperparameters and the definition of the loss. In contrast, NNs can learn complex functions having many hyperparameters under a large training dataset but are difficult to integrate into a machine-driven experimental loop with limited initial data and expensive evaluations, especially at the early stage of sampling. Hence, they proposed a two-step framework that combines the optimization assets of BO with the regression ability of Deep Neural Network (DNN).

## 3. Applications of Microreactor as a Discovery Accelerator

### 3.1. High-Throughput Screening and Optimization

Microreactor technologies have been applied to implement automated experimentation for high-throughput screening and optimization of chemical reactions in continuous flow. The integration of machine learning algorithms and closed-loop flow control in automated experimentation enables efficient exploration of experimental parameters. Researchers have extensively investigated the effectiveness of these systems for various types of chemical reactions, demonstrating the inherent advantages of microreactors over bulk systems in terms of throughput, cost, material consumption, and product quality.

Photo-redox catalysis is an important class of catalytic reaction, particularly in biomedical and materials science, as it offers unique chemical reactivity under relatively mild conditions [54]. To enable high-throughput screening of photocatalytic reactions, Coley et al. have developed an automated flow experiments platform based on a modular flow reactor system [55]. This system incorporates various modular components, including a liquid handler for sampling a photocatalyst library, a horseshoe-shaped microreactor that can be configured for oscillatory or continuous flow mode, a fiber-coupled photo-detection unit, and online LC/MS analyzers. The microreactor encapsulated in an aluminum block allows for an accurate and systematic study of the effect of reaction temperatures. The oscillatory flow path allows flexible and precise control of residence time while facilitating efficient mixing, rapid heat transfer, and uniform photo-illumination across the reaction volume. The reaction product can be analyzed subsequently by a fiber-coupled UV-Vis spectrometer and online LC/MS analyzers. Using this setup, they screened optimal reaction conditions using only 15 µL of the reaction mixture per experiment, with a total volume of 4.5 mL to screen 150 reaction conditions. The same system is then configured for continuous synthesis mode at a total flow rate of 250 µL/min.

Electroorganic chemistry presents a promising venue for sustainable chemical transformation. Implementing electrochemistry in microfluidics devices is particularly advantageous because of the physical arrangement of the electrodes interfaced with the reaction volume. The large surface-to-volume ratio and the improved cell conductivity can increase the reaction rate and consequently improve the efficiency of screening and optimization experiments. Small physical dimensions of the reaction field can also provide improved control of the reaction conditions. Mo et al. developed a microreactor flow synthesis and analytical platform for single-electron transfer redox-neutral chemistry [56]. Their reactor featured an electrochemical flow cell equipped with novel interdigitated electrodes. This reactor design allowed for a very rapid exchange of reactive intermediates within a mere 100 ms. To investigate the influence of reaction time and potential on product yields, an inline HPLC system was incorporated. Furthermore, by configuring the setup into a microliter-scale cyclic voltammetry (CV) module, the authors demonstrated rapid and material-efficient kinetic measurements. In the high-throughput screening mode, an optimal range of potential is determined within only 10 h of run time and using 300 µL of reagents, saving considerable time and materials compared to manual screening. Rapid measurement of kinetics for two important mediated anodic oxidations is achieved using the platform in the microliter-scale CV flow cell mode.

**Figure 5:**
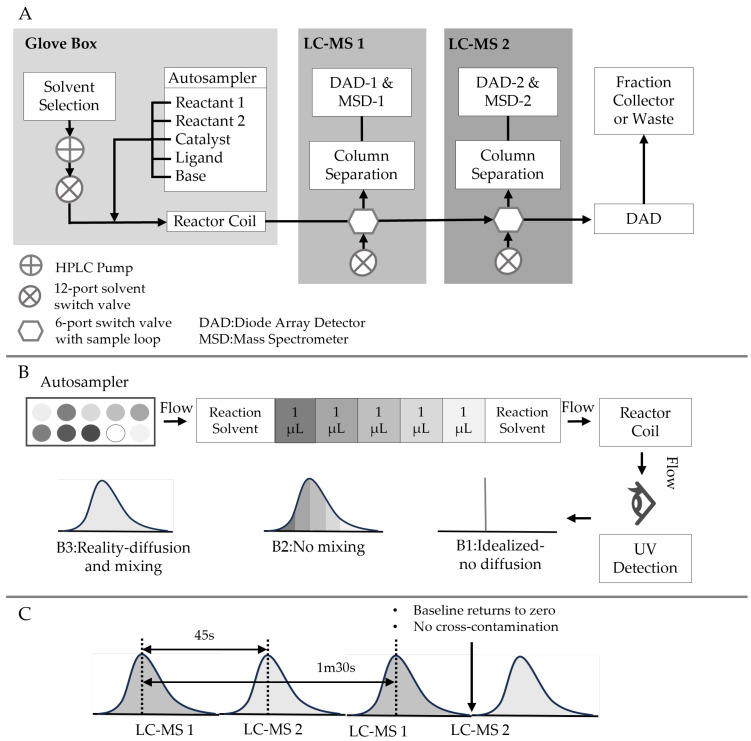
Flow system setup and segment preparation. (**A**) Schematic depiction of the flow system. (**B**) Segment preparation and injection into flow stream showing potential mixing and diffusion outcomes, (**B1**) idealized–no diffusion, (**B2**) no mixing, and (**B3**) observed. (**C**) Portrayal of UV trace of the emerging reactions’ segments and fractionation into alternating LC-MS units.

The demand for cost-effective high-throughput screening is particularly high in the early stage of drug development campaigns. Perera et al. introduced an automated flow synthesis platform that utilizes commercially available components and modified inline HPLCs with an automated well-plate sampler [57] (Figure 5). This microreactor system was validated through a model Suzuki-Miyaura cross-coupling reaction. The automated flow reactor efficiently handled sample preparation and product analysis of up to 1500 reaction segments within 24 h, consuming only tens of nanomoles of substrate per reaction. It enabled extensive exploration of a wide range of reaction parameters while minimizing experimental time and material consumption. The optimal reaction parameters identified in the exploration mode were subsequently replicated using the same platform configured in the production mode, achieving product yields at the hundreds of milligrams scale.

Expanding the applicability of microreactors to broader chemical reactions and material synthesis is an active area of research. Ongoing research efforts by both academia and industry have yielded a substantial body of evidence showcasing the successful application of microreactor systems in a high-throughput screening and optimization campaign for the synthesis of small organic compounds, polymers, and nanomaterials.

### 3.2. Automated Material Synthesis in Continuous Flow

**Figure 6:**
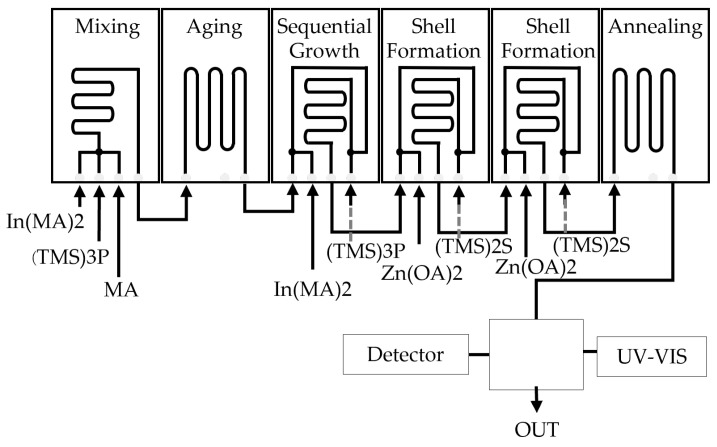
Multistage microfluidic platform for the synthesis of InP/ZnS core/shell QDs. The first three stages (mixing, aging, and sequential growth reactors) are used for the synthesis of InP cores and the following three stages (two shell formation reactors and one annealing reactor) for the synthesis core/shell morphologies.

Microreactors have found applications in the synthesis of inorganic nanomaterials and polymers. Despite extensive studies on the flow synthesis of nanocrystals with a homogeneous structure, few studies have been reported on the preparation of heterogeneous structures, such as core-shell morphologies, in flow systems. This can be attributed to the challenge of achieving slow additions of overcoating precursors to prevent secondary nucleation of the shell material, which is difficult to accomplish using tubular reactors or droplet-based systems. To address this limitation, Baek et al. developed a multi-staged microreactor for the synthesis of quantum dots (QDs) in flow [58] (Figure 6). Their multistage silicon-pyrex on-chip reactor facilitates precise control of high-temperature profiles and high-pressure flow distribution across microfluidic channels, with up to 6 stages available for the synthesis of various III-V-based core/shell QDs. The sub-channels in the shell-growth microreactors maintain a low concentration of shell precursors, effectively suppressing undesirable secondary nucleation. Another notable application of microreactor technologies was demonstrated by Ergorov et al., who employed them for the synthesis of gold nanoparticles (AuNPs) with various form factors for nano-medicine applications [59]. The chemical and physical characteristics of AuNPs are critically determined by factors such as size, surface morphology, and charges. Even subtle changes in the formulation process and composition can significantly impact the properties of AuNPs, leading to variable medicinal effects and unwanted consequences. Therefore, precise control of the formulation process and composition is essential for better management of these factors. The use of microreactors has been proven effective in precisely controlling polymerization conditions for polymer synthesis. In polymer synthesis, the ability to control the molecular weight distribution plays a crucial role in achieving the desired material properties. Iwasaki et al. demonstrated control of the molecular weight distribution in free radical polymerization using microreactors, which exhibited significantly higher heat removal efficiency compared to batch reactors [60]. They found that microreactors are highly effective in controlling the molecular weight distribution for highly exothermic free radical polymerizations, such as butyl acrylate (BA), benzyl methacrylate (BMA), and methyl methacrylate (MMA), but their effectiveness is limited for less exothermic polymerizations, such as vinyl benzoate (VBz) and styrene (St). Similar studies were conducted by Wenn et al., who explored the control of the molecular weight distribution in PhotoRAFT (reversible addition fragmentation radical transfer) polymerization [61]. Additionally, Rizkin et al. have demonstrated the use of a microreactor system combined with machine learning algorithms to design efficient polymerization reactions [9]. The authors developed an integrated continuous-flow microfluidic platform that combined pumps, manifolds, controls, and analytics, enabling automated and high-speed experimentation. In-situ infrared thermography was integrated into the system to capture exotherm data, which are closely related to catalytic productivity. To analyze the productivity data, the authors employed ANN to fit the data, achieving an average error of 0.498 percent when compared to experimentally obtained catalytic productivity. Utilizing the ANN model of the reaction space, the authors were able to significantly reduce the number of experiments conducted and the amount of chemical waste generated while reliably extracting fundamental kinetic parameters.

### 3.3. Rapid and Precise Measurement of Reaction Kinetics

Precise knowledge of reaction kinetics is essential for the development of greener, safer, and more economical chemical processes. Extracting precise kinetic parameters and models is a crucial step for designing and optimizing process flow and reactor configurations. The significance of acquiring precise kinetic information has been further emphasized with the recent integration of machine learning techniques in the optimization of chemical reactions, where the quality of the kinetic data directly influences the performance of the machine learning models. In kinetic experiments, the kinetic parameters are typically obtained by fitting a kinetic model to the temporal profile of the reaction dynamics. While batch reactors provide access to multiple temporal data points in a single experiment, they are susceptible to slow mixing and substantial experimental variability due to uncontrollable factors, such as temperature inhomogeneity across the reaction volume. This often leads to inaccurate determination of kinetic models and parameters. In contrast, microreactor environments facilitate rapid mixing, on the order of microseconds, and well-defined reaction fields that enable precise control and knowledge of the temperature profile and residence time across the reaction volume. This enhanced control and accuracy make a microreactor advantageous for extracting reliable kinetic information.

**Figure 7:**
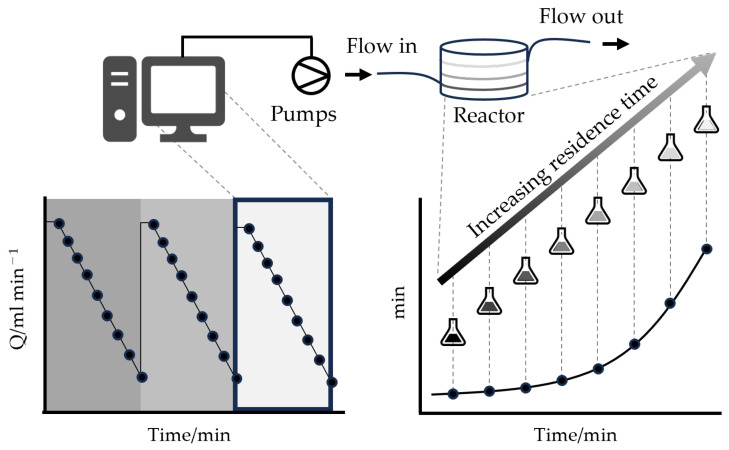
A representation of how linear gradient flow ramps can be utilized to sample with a high data density on the initial curvature of the kinetic plot.

The advantages of microreactors in rapidly determining kinetic information were initially demonstrated by Mozharov et al. using the Knoevenagel condensation reaction [62]. Their approach involved a step change in flow rate and real-time noninvasive Raman measurement at the end of the flow line, allowing extraction of the location-specific kinetic information without the need to move the measurement probe along the microreactor channel. However, this method faced practical challenges, such as the non-instantaneous response of the flow rate, requiring graphical and semi-empirical fitting to the data. To overcome this, Moore et al. introduced a controlled ramp that defined the system’s instantaneous residence time in transient, eliminating the need to wait for the system to reach a steady state (Figure 7) [63]. This “pseudo-batch” approach, or transient flow experiment, treated each segment of the continuous flow system as a distinct batch reactor with a specific residence time. This approach allowed a higher sampling rate and reduced error in estimating kinetic parameters. Subsequently, Hone et al. demonstrated the generation of kinetic models for multistep reactions using data obtained in transient flow experiments [64]. Further extending the capabilities of transient flow experiments, Fath et al. incorporated inline FT-IR spectroscopy for real-time experiments, enabling rapid acquisition of kinetic data and determination of the kinetic models [65]. Multidimensional transient experiments involving the simultaneous variation of more than two reaction conditions led to further reductions in experiment run-time and material consumption [66,67]. More recently, Taylor et al. introduced automated model identification techniques based on machine learning algorithms [68,69]. This technique only requires information about the participating chemical species (i.e., starting materials, intermediates, and products) along with transient experiment data. By employing machine learning algorithms to assess kinetic models generated through chemical reaction network analysis and fit parameters, the authors successfully mitigated human bias and achieved enhanced model prediction. The transient flow technique can be potentially applied to a wide range of chemistry, including photochemical reactions [70].

In addition to the automated model identification technique based on machine learning described above, as a measure to further improve real-time analysis, a digital twinning (DT) approach to flow-type chemical reaction systems has been initiated by assimilating experimental flow-type chemical reaction systems with mathematical models of fluid and chemical reactions that theoretically simulate these systems. This approach uses various state information (e.g., pressure and temperature) obtained from the flow reaction system and observed information on the products of reactions as boundary conditions to sequentially estimate unknown parameters, such as reaction rate constants given in the mathematical model. By using DT technology with high real-time performance, it is expected to be possible to quickly estimate constant values from relationships in mathematical models based on a vast amount of state and observation data obtained in time series, even when multiple and complex parameters are to be estimated. Furthermore, in sequential and equilibrium reactions, the ability to extend the sequential reaction field in the flow direction and to control equilibrium by continuous separation and removal of reaction products from the system is expected to further reduce noise in the chemical process and enable the search for pure reaction pathways.

The author’s group has also recognized the usefulness of this approach and is constructing a DT infrastructure for flow-type chemical reaction systems. The mathematical model of the fluid/chemical reaction, input/output functions, and estimation calculation functions are all included in the same model hierarchy to improve consistency with the hardware and increase the speed of assimilation calculations. The search conditions set in the mathematical model, such as the addition rate of raw materials and catalysts and the temperature of the reaction field, are converted into the pump operation and the applied signal to the heater in a real experimental system via the input/output interface of the PC. On the other hand, the resulting state data for the reaction field, such as temperature and pressure, and the observed data for reaction products are converted into digital signals via the same interface and returned to the mathematical model. Parameter values are learned and estimated from the comparison of predicted and measured values.

## 4. Toward Democratizing Microreactor Technology for Accelerated Discoveries

### 4.1. Standardization for Microreactor Technologies for AD

Setting standardized protocols and methodologies is essential for the broader adoption and reproducibility of microreactors in flow chemistry. While remarkable advancements in both hardware and software have been made in this field, and many components are now available commercially as plug-and-play components, there has been little focus on establishing standards among researchers and developers. Consequently, potential users, especially novice ones, face great challenges in adapting and incorporating technological advancements made by others in the field. In the era of accelerated discovery, where scientific and technological advancements are expected to occur at a much higher rate than ever before, it is crucial to develop standardized practices and guidelines to foster collective research and development. There are several aspects of microreactor technologies, and each needs advancements to be made toward setting standards. Hone et al. argued the importance of describing the details of parameters and protocols involved in the design of experimental setup, flow experiments, and data analysis as they interdependently affect experimental outcomes and their interpretations. They provided guidelines for hardware design and handling, parameter controls in flow, data processing and sharing, and other best practices in laboratories to ensure reproducibility, scalability, and reliability [71].

Establishing standards in fabrication processes is an essential step towards digitally connected fabrication processes. By achieving digitally connected fabrication processes, researchers can streamline the production of microreactor devices for flow synthesis. This approach enables efficient design translation, automation of fabrication steps, real-time quality control, and data-driven optimization.

**Figure 8:**
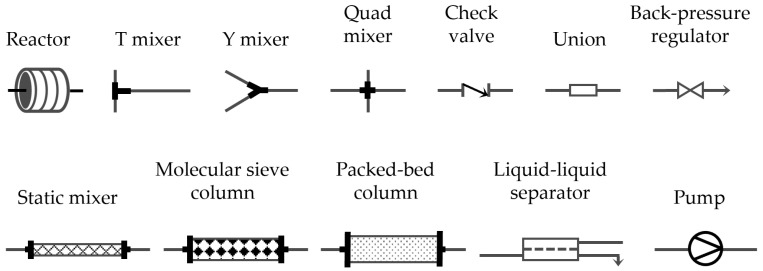
Typical components used in the assembly of continuous flows.

Characterizing microreactor devices is crucial for understanding their performance and optimizing their operation. Standardizing characterization methods will ensure evaluation and enable comparisons between different experiments using different platforms (Figure 8).

Standardizing operation procedures is also crucial for broader adoption and implementation. Operations such as temperature control, pressure regulation, and flow control need to be carefully defined and optimized to ensure reproducibility, scalability, and efficiency. In automation and optimization, standardized operation procedures provide a framework for optimizing and fine-tuning reaction conditions. By systematically varying key parameters within defined ranges, researchers can identify optimal conditions that maximize yield, selectivity, and reaction rate.

Safety standards are another important aspect of technology standardization. By clearly defining safety protocols, researchers can minimize the risks associated with handling hazardous or reactive materials. Standardized safety procedures ensure operators, be they humans or robots, follow proper environmental and safety guidelines.

### 4.2. Integrated Development Environment of Microreactor Development

While microreactor technologies have the potential to significantly expedite the experimental validation in an accelerated discovery campaign, the development of a microreactor system is itself typically a resource-intensive and time-consuming endeavor, particularly when microreactor design and hardware architecture require iterative optimization. In order to streamline the development process of microreactors, the concept of an Integrated Development Environment (IDE), which has been developed and become indispensable in the semiconductor industry, can be adopted. An IDE provides a comprehensive framework that encompasses all stages of microreactor development, from design to validation. Such an integrated framework will enable rapid implementation of validation experiments and alleviate the bottleneck in accelerated campaigns.

There are several key requirements for the realization of an IDE for microreactor development. Developing an IDE requires the seamless integration of various components, including software algorithms, hardware interfaces, data acquisition systems, and control mechanisms. The complexity arises from the need to connect diverse elements and ensure their compatibility and interoperability within the IDE framework. Unlike electric circuits, which can be decomposed into fundamental elements such as resistors, capacitors, and transistors, there is no standardized method to decompose fluidic channels into elements whose aggregated behavior can be systematically computed. As a result, researchers have developed and adopted different technologies and approaches that accommodate their research requirements. Achieving compatibility across different microreactor platforms, software tools, and experimental setups can be a significant challenge due to the heterogeneity of technologies and approaches. Establishing standardized protocols, formats, and interfaces is therefore crucial to enable the smooth exchange of data and information between different modules within the IDE. An IDE should also be designed to accommodate a wide range of microreactor configurations, experimental setups, and research objectives. It should be scalable and flexible to support different scales and complexities of microreactor systems, allowing researchers to adapt and customize the IDE to their specific requirements. Efficient data management, storage, and analysis are fundamental to the success of an IDE. Handling large volumes of experimental data generated by high-throughput experiments and integrating them with machine learning algorithms and data analytics tools pose challenges in terms of data organization, quality control, and real-time processing capabilities. Designing an IDE that is user-friendly and accessible to researchers with varying levels of technical expertise is another key requirement for its widespread adoption. Lastly, the development of such an IDE with these characteristics requires collaborative efforts between researchers, hardware engineers, software developers, system architects, and designers.

**Figure 9:**
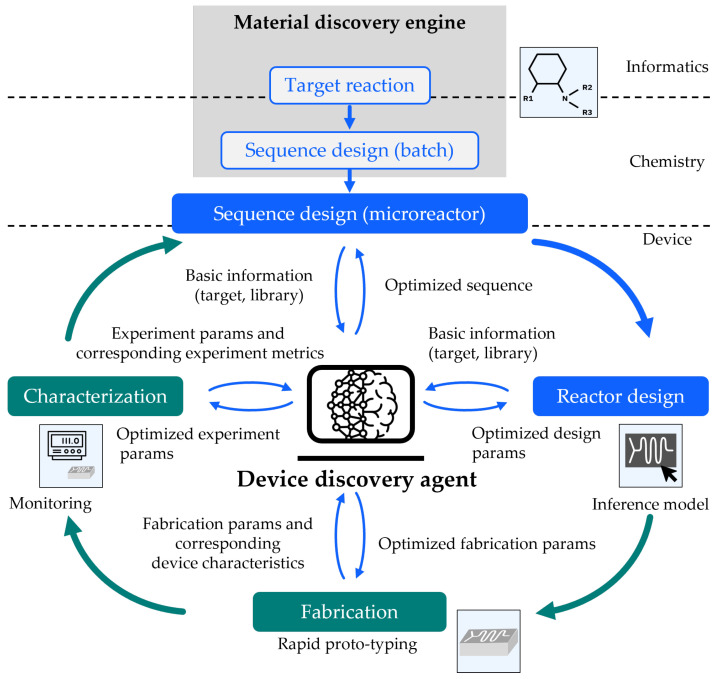
Workflow for IDE and device discovery agent. On Insight Monitoring [32,33,34], Outside Monitoring [28,35,36,37,38], Rapid proto-typing [26,27,28,29], and Inference model [39,40,41,42,43,44,45,46,47,48,49] are cited.

Figure 9 represents a comprehensive workflow for microreactor development, encompassing reaction sequence design, reactor design, fabrication, and characterization stages. The reaction sequence design phase involves determining a suitable action sequence for the chemical processes within the microreactor system, derived from batch reaction knowledge using a hypothesis generator. Reactor design follows, utilizing simulation tools or custom software to derive a reactor system design, including device specifications and properties. In the fabrication phase, microfluidic components are produced, with increasing automation potential for future robotic handling systems. The subsequent characterization phase involves experimental evaluation of the reactor system, measuring chemical reaction metrics and comparing them to predetermined criteria. This iterative workflow continues until the desired results are achieved. To achieve this procedure, the device discovery agent shown in the center of the figure plays a critical role by seamlessly connecting these phases, minimizing manual data processing, and is a key component to be established.

### 4.3. Democratizing Microreactors Technology by Open-Source Software and Platforms

The development of open-source software and platforms for microreactor technologies is of paramount importance for their widespread adoption. Microreactor technologies present numerous advantages for process automation, optimization, and autonomous experiments. However, building an autonomous microreactor system can be challenging because the system contains many hardware and software components that need to be reconfigured and reconnected for different processes and experiments. The successful implementation of an autonomous microreactor system requires expertise and proficiency across various scientific and engineering domains. Open-source software and platforms foster collaboration and knowledge sharing among researchers and engineers with various technical backgrounds and research purposes. By providing a shared space for data, software, and hardware tools, practitioners can collaborate on the design, implementation, and characterization of microreactor technologies, accelerating their development and scientific discoveries and innovation through their use.

Despite the significant advantages that open innovation for the development of microreactor systems offers to the research community, the development of open-source platforms in this field is still in its early stages. LevyLabis is a great example of an open platform for flow chemistry automation, providing uses of various technical levels with the ability to monitor and control chemical reactions remotely through the Internet [72]. The platform enables the automation of synthetic procedures and self-optimization of reaction parameters, demonstrating multiobjective optimization tasks to maximize reaction product output. OpenFlowChem is another excellent example of an open platform for flow chemistry automation, designed for creating flexible control and automation systems with quick deployment and modifications [73]. The authors demonstrated its capability for rapidly reconfiguring systems for three different hydrogenation reactions in flow. Open-source sharing of microreactor design has been even slower compared to software sharing. Metafluidics is an open-source repository that hosts digital design files, assembly specifications, and software for building an operating microreactor for synthetic biology [74]. The goal of Metafluidics is to engage a wide community of individuals, including engineers and DIY enthumssiasts, in microfluidic research, even with limited fabrication skills. A similar open-source software tailored for flow microreactors can stimulate participation from researchers from multiple disciplines, encouraging them to contribute their expertise, share resources, and collaborate on improving the platform to drive innovation in microreactor technologies.

## 5. Conclusions

This survey paper provides an in-depth review of recent advancements in microreactor technologies, focusing on their role in facilitating accelerated discoveries in chemistry and materials. After investigating the applicability of microreactor-enabled discovery accelerators in chemical and materials contexts, we found that the integration of machine intelligence and automation into microreactor-based experiments is a fully integrated automation strategy. There have been challenges in establishing intelligent systems. We reviewed new technologies that can help facilitate the implementation of microreactor-enabled discovery accelerators to address this issue. Then, we proposed an advanced integrated microreactor development platform with a device discovery agent that can handle microreactor design, workflow, sequence design, etc., in an integrated manner.

## Figures and Tables

**Table 1:** Fabrication technologies for microreactor.

Method	Material	Resolution	Application	Characteristics	Ref.
Lithography and etching techniques	Silicon, Glass	100 μm~100 nm	Fine flow channels	Compatibility with semiconductor technology, chemical resistance, heat resistance, robustness	[31]
Imprint molding	Silicone, Glass	100 μm~10 μm	Chemical reaction, Chemical analysis	Resin mold, metallic mold, optical transparency	[32]
Precision machining techniques	Glass, Metal	1 mm~10 μm	Chemical reaction	Corrosion resistance, thermal conductivity, chemical resistance, heat resistance, robustness	[33]
3D printing technique	Resin, Metal	1 mm~10 μm	Packaging	3D structures	[34,35,36]

**Table 2:** Reaction monitoring.

Deployment	Method	Monitoring Targets	Ref.
In situ	Electrochemical detection	Gas, Liquid	[37,38]
	TG-TEM *	Inorganic, metallic nanoparticles	[39]
Detection from outside	X-ray absorption	Metallic nanoparticles	[40]
	NMR	Organic materials	[41,42,43]
	Optical detection	Organic materials	[44]

* TG-TEM: thermogravimetric−transmission electron microscopy.

## Data Availability

No new data were created or analyzed in this study. Data sharing is not applicable to this article.
